# A 10-day vacancy period after cleaning and disinfection has no effect on the bacterial load in pig nursery units

**DOI:** 10.1186/s12917-016-0850-1

**Published:** 2016-10-19

**Authors:** K. Luyckx, S. Millet, S. Van Weyenberg, L. Herman, M. Heyndrickx, J. Dewulf, K. De Reu

**Affiliations:** 1 Institute for Agricultural and Fisheries Research (ILVO), Merelbeke, Belgium; 2 Department of Pathology, Bacteriology and Poultry Diseases, Ghent University, Faculty of Veterinary Medicine, Merelbeke, Belgium; 3 Veterinary Epidemiology Unit, Department of Reproduction, Obstetrics and Herd Health, Faculty of Veterinary Medicine, Ghent University, Merelbeke, Belgium

**Keywords:** Nursery pigs, Between batch vacancy, Cleaning and disinfection, Bacterial load

## Abstract

**Background:**

Biosecurity measures such as cleaning, disinfection and a vacancy period between production cycles on pig farms are essential to prevent disease outbreaks. No studies have tested the effect of a longer vacancy period on bacterial load in nursery units.

**Methods:**

The present study evaluated the effect of a 10-day vacancy period in pig nursery units on total aerobic flora, *Enterococcus* spp., *Escherichia coli*, faecal coliforms and methicillin resistant *Staphylococcus aureus* (MRSA). Three vacancy periods of 10 days were monitored, each time applied in 3 units. The microbiological load was measured before disinfection and at 1, 4, 7 and 10 days after disinfection.

**Results:**

No significant decrease or increase in *E. coli*, faecal coliforms, MRSA and *Enterococcus* spp. was noticed. Total aerobic flora counts were the lowest on day 4 after disinfection (i.e. 4.07 log CFU/625 cm^2^) (*P* < 0.05), but the difference with other sampling moments was limited (i.e. 0.6 log CFU/625 cm^2^) and therefore negligible. Furthermore, this observation on day 4 was not confirmed for the other microbiological parameters. After disinfection, drinking nipples were still mostly contaminated with total aerobic flora (i.e. 5.32 log CFU/625 cm^2^) and *Enterococcus* spp. (i.e. 95 % of the samples were positive) (*P* < 0.01); the feeding troughs were the cleanest location (total aerobic flora: 3.53 log CFU/625 cm^2^ and *Enterococcus* spp.: 50 % positive samples) (*P* < 0.01).

**Conclusions:**

This study indicates that prolonging the vacancy period in nursery units to 10 days after disinfection with no extra biosecurity measures has no impact on the environmental load of total aerobic flora, *E. coli*, faecal coliforms, MRSA and *Enterococcus* spp..

## Background

Weaned piglets are subjected to many environmental, behavioural and dietary stresses. Moreover, the intestinal gut flora is still precarious, which makes them highly susceptible to enteric diseases [1]. Disease outbreaks in animal houses can lead to animal mortality and higher condemnation rates at slaughterhouses. The resulting economic damage can be severe [2] together with preventive measures (e.g. quarantine in case of epidemics) and even destruction of farm animals [3]. In addition, foodborne zoonotic diseases are a significant and widespread global public health threat.

In nursery units, diarrhoea is one of the most important causes of economic losses in the pig industry. Post-weaning diarrhoea is multifactorial but the proliferation of pathogenic *Escherichia coli* strains throughout the intestinal tract of piglets after weaning has been shown to play a significant role [4, 5]. Another important pathogen for the pig industry is *Salmonella*. In 2011, most of the reported food-borne outbreaks (69 553 human cases) in the European Union were associated with food originating from animals. *Salmonella* was the most frequently detected causative agent (26.6 % of outbreaks) [6].

Methicillin resistant *Staphylococcus aureus* sequence type 398 (MRSA ST398) is an emerging opportunistic pathogen among farm animals, especially pigs [7–9]. Epidemiological studies have shown that they not only colonise pigs, but can also be transmitted to persons with direct livestock exposure. Moreover, it is indicated that MRSA ST6398 represents an increasing cause of infections in humans [10].

It is of great importance to prevent disease outbreaks through biosecurity measures rather than cure them [3]. Biosecurity includes all measures that prevent pathogens from entering a herd (external biosecurity) as well as reducing the spread of pathogens within the herd (internal biosecurity) [11]. Between production cycles, internal biosecurity measures such as cleaning, disinfection and a vacancy period are applied. Every biosecurity measure can influence the degree of infection pressure before new animals arrive.

Luyckx et al. [12] showed that a cleaning step in broiler houses caused a reduction of total aerobic flora by 2 log CFU/625 cm^2^ and that a disinfection step caused a further reduction of 1.5 log CFU. In piglet nursery units, the importance of a prolonged vacancy period is unknown. The aim of the present study was to assess the evolution of the bacterial load of total aerobic flora, *Enterococcus* spp., *E. coli*, faecal coliforms and MRSA during a 10-day vacancy period in piglet nursery units. *Enterococcus* spp. and faecal coliforms are suggested to be adequate hygiene-indicator organisms for faecal contamination of surfaces. In addition, *E. coli* have been shown to be suitable index organisms for monitoring the possible presence of *Salmonella* [13–15].

## Results

**Fig. 1:**
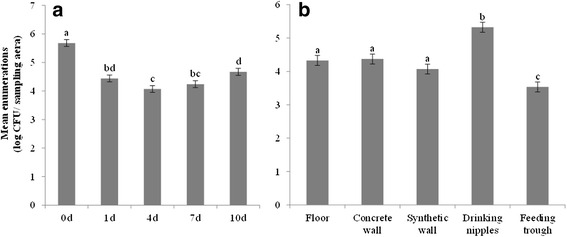
Mean enumeration of total aerobic flora with standard errors. Mean enumerations are given for each sampling moment (**a**) and location after disinfection (**b**). Samples (*n* = 135) were taken before disinfection (0d) and 1 day (1d), 4 days (4d), 7 days (7d) and 10 days (10d) after disinfection. Samples (*n* = 108) were taken from each location. Significant differences between sampling moments/ locations are indicated by different letters above bars

**Fig. 2:**
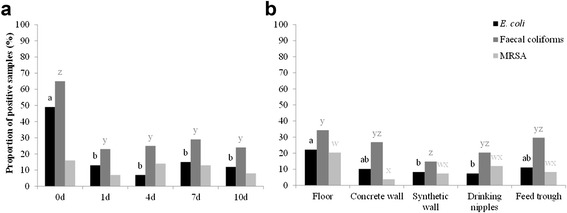
Proportion of positive samples given for detection of *E. coli*, faecal coliforms and MRSA, respectively. Proportions are given for each sampling moment (**a**) and location after disinfection (**b**), in percentage. Samples (*n* = 135) were taken before disinfection (0d) and 1 day (1d), 4 days (4d), 7 days (7d) and 10 days (10d) after disinfection. Samples (*n* = 108) were taken from each location. Significant differences between sampling moments/ locations per bacteriological parameter are indicated by different letters above bars

**Fig. 3:**
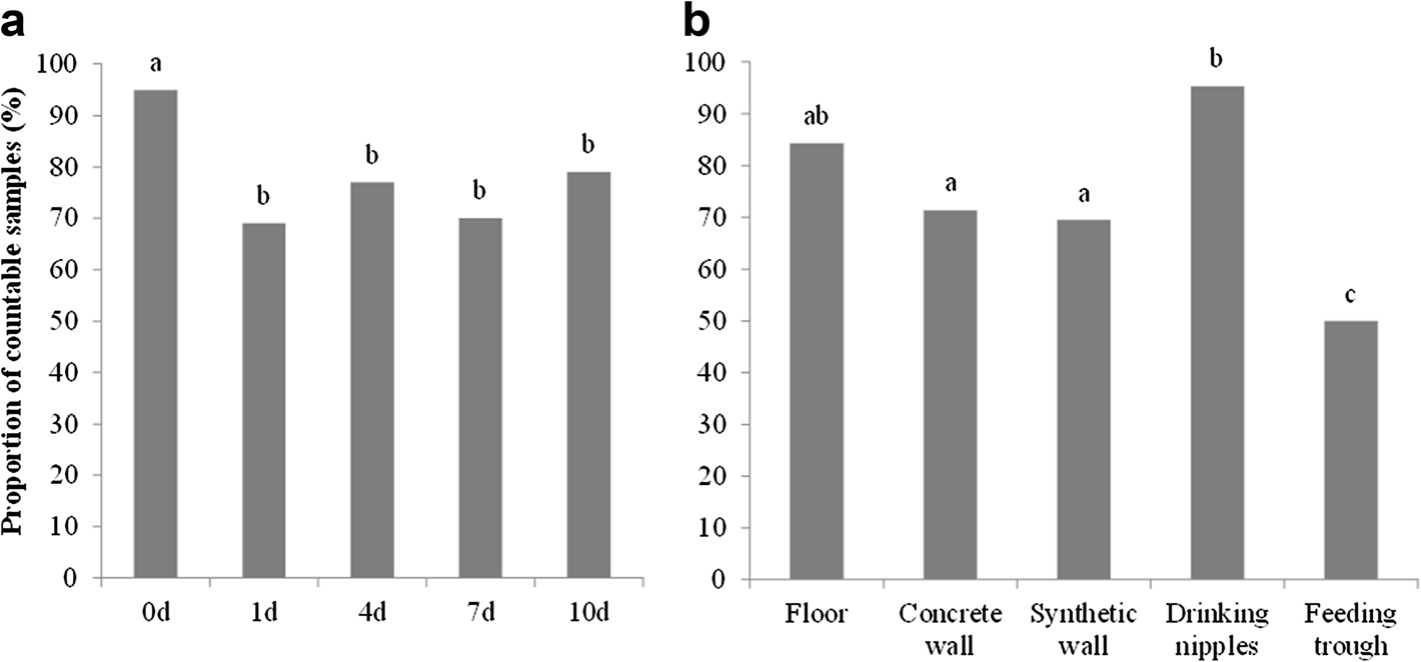
Proportion of countable samples given in percentage for *Enterococcus* spp.. Proportions are given for each sampling moment (**a**) and location after disinfection (**b**). Samples (*n* = 135) were taken before disinfection (0d) and 1 day (1d), 4 days (4d), 7 days (7d) and 10 days (10d) after disinfection. Samples (n =108) were taken from each location. Significant differences between sampling moments are indicated by different letters above bars

Before disinfection, the mean enumeration of total aerobic flora was 5.64 log CFU/625 cm^2^ (Fig. 1a). The proportion of positive samples for *E. coli*, faecal coliforms and MRSA (after enrichment) and *Enterococcus* spp. was 49, 65 and 16 % (Fig. 2a) and 95 % (Fig. 3a), respectively.

On day 1 after disinfection, mean enumeration of total aerobic flora was significantly reduced to 4.44 log CFU/625 cm^2^ (*P* < 0.01) (Fig. 1a). Of the 135 samples taken on day 1, 13, 23 and 7 % were positive for *E. coli*, faecal coliforms and MRSA detection, respectively (Fig. 2a). In addition, 69 % of the samples gave countable results for *Enterococcus* spp. (Fig. 3a). The proportion of positive samples for *E. coli*, faecal coliforms and *Enterococcus* spp. was significantly lower compared to the proportions found before disinfection (*P* < 0.01).

Three days later (day 4), total aerobic flora were significantly reduced to 4.07 log CFU/cm^2^ (*P* < 0.05). Only 7 % of the samples were positive for *E. coli*, but the number of positive samples found for faecal coliforms and MRSA were higher (25 and 14 %, respectively). Countable results for *Enterococcus* spp. also increased to 77 %.

On day 7 after disinfection, mean enumeration of total aerobic flora was 4.24 log CFU/625 cm^2^. Of all samples, 15, 29 and 13 % were positive for *E. coli*, faecal coliforms and MRSA detection, respectively and comparable to day 1, 70 % of the samples gave countable results for *Enterococcus* spp..

On day 10, total aerobic flora increased further to 4.67 log CFU/625 cm^2^, which was 0.6 log CFU more than 4 days after disinfection (*P* < 0.01), but not significantly different from day 1. Proportion of positive samples for *E. coli*, faecal coliforms and MRSA were 12, 24 and 8 %, respectively. In addition, 79 % of the samples were countable for *Enterococcus* spp..

Overall, no significant differences were noticed between sampling moments after disinfection for *E. coli*, faecal coliforms, MRSA and *Enterococcus* spp..

During the entire 10-day vacancy period, the overall contamination level (total aerobic flora) was the highest for drinking nipples (i.e. 5.32 log CFU/625 cm^2^) (*P <* 0.01) and the lowest for feeding troughs (i.e. 3.53 log CFU/625 cm^2^) (*P <* 0.01) (Fig. 1b). Results of *Enterococcus* spp. confirmed these observations (*P* < 0.01) and also showed that the floors were still highly contaminated (i.e. still 84 % of the samples were positive) (*P* < 0.01) (Fig. 3b). Results for *E. coli*, faecal coliforms and MRSA did not indicate the most critical locations after cleaning and disinfection (C&D) (Fig. 2b).

**Fig. 4:**
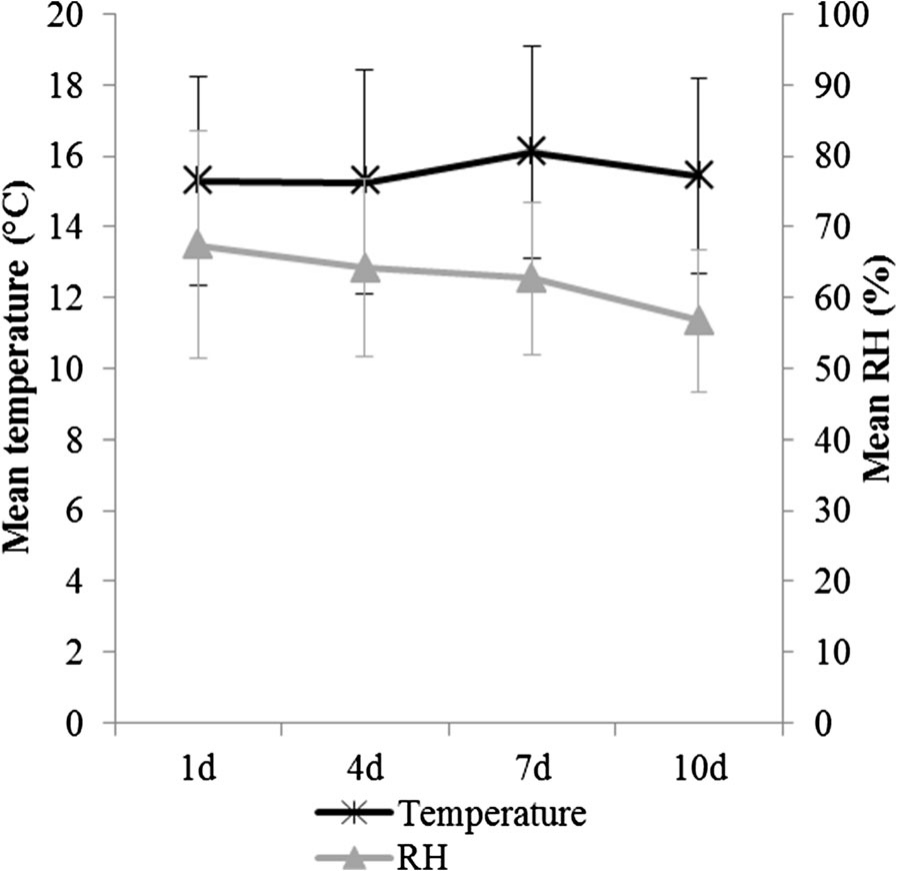
Mean temperature (°C) and relative humidity (RH, %) with standard deviations given per sampling moment. Sampling moments: day 1 (1d), 4 (4d), 7 (7d), 10 (10d) after disinfection

During the vacancy period, mean temperature ranged from 15 °C to 16 °C and relative humidity (RH) from 57 to 67 % (Fig. 4). These two parameters did not have a significant effect on the different bacteriological parameters.

## Discussion

Biosecurity measures, such as cleaning and disinfection (C&D) and a prolonged vacancy period of the animal houses are an essential part of the hygiene management on the farm to prevent disease outbreaks. The effect of a vacancy period of 10 days after disinfection on several bacteriological parameters was examined during this study.

Disinfection reduced the total aerobic flora by 1.2 log CFU/ sampling surface. During the following 10 day vacancy, only a small reduction was observed on day 4, though this seemed microbiological negligible (maximum difference of 0.6 log CFU/625 cm^2^). One possible explanation for the observed small fluctuations and the decline of total aerobic flora on day 4 is that some bacteria can survive stressful conditions by entering a viable but nonculturable state [14]. These nonculturable bacteria were not enumerated nor detected by the methods used in this study. Another possible explanation is that residual flora could proliferate again after disinfection, due to lack of niche and nutrient competition with other bacteria. These residual bacteria could have survived the disinfection step by the presence of a resistance mechanism [16–19] or by detrimental factors present during disinfection, such as residual organic material.

Moreover, a longer vacancy period can even have a negative effect, not only financially because of a lower number of production cycles (i.e. lower income) but also bacteriologically. For example, recontamination could occur by vectors such as vermin and rodents in case of biosecurity breaches [20–22], especially when other compartments in the same building are still filled with animals or if residual organic material (e.g. faeces and feed) is present after C&D. Flies may be reservoirs and vectors of several bacteria such as *Salmonella* [20, 23, 24]*, E. coli* O157:H7 [25], *Staphylococcus aureus* [26] and *Streptococcus suis* type 2 [27]. Wild rodents can also carry pathogens such as *Salmonella, Campylobacter, Yersinia* and MRSA ST398 [28–31]. As biosecurity measures are very well implemented on the pilot farm, it can be assumed that on other farms, the bacteriological load and infection pressure may even increase during vacancy.

Some bacteria can survive for long periods under various conditions in the environment, such as *Salmonella*, *Staphylococcus aureus* (including MRSA) and *Enterococcus* spp. [32]. The results from the present study indicate that a prolonged vacancy period without extra biosecurity measures creates no reduction in these bacteria. Extra biosecurity measures such as specific pathogen control programs and pest control during the vacancy period could therefore be beneficial.

Finally, the contamination levels of several locations were analysed during the vacancy period. Drinking nipples were still mostly contaminated with total aerobic flora and *Enterococcus* spp.. Luyckx et al. [33] showed that drinking cups are critical locations for C&D in broiler houses. Drinking water from these contaminated sources could be a possible cause for disease in animals. Therefore extra attention should be given to these locations during C&D and during the vacancy period. In addition, also disinfection of drinking lines is recommended as they can be contaminated with biofilms, including pathogenic bacteria [34]. As this study is carried out on an experimental farm, also other locations can be identified as critical locations for C&D, due to their different specific structural design or composition compared to the studied farm.

## Conclusion

This study indicates that a vacancy period up to 10 days after cleaning and disinfection with no extra biosecurity measures has no beneficial effect on the bacterial load of total aerobic flora, *E. coli*, faecal coliforms, MRSA and *Enterococcus* spp. in piglet nursery units.

## Methods

### Sampling plan

This study was carried out in 6 identical nursery units (A1 to A3 and B1 to B3) on the experimental pig farm at the Institute for Agricultural and Fisheries Research (ILVO, Merelbeke, Belgium). Each unit consists of 8 pens of 1.8 m^2^. Piglets were moved to these units immediately after weaning (4 weeks of age) and stayed there for 6 weeks. Each pen housed 6 piglets. Pen flooring was a synthetic grid, under which a board slopes towards a centrally-located slurry pit. Units A1 to A3 were monitored during 2 successive vacancy periods in February and April 2015 and units B1 to B3 during 1 vacancy period in March 2015. After pig removal, units were soaked with water. The day after, units were cleaned with hot water (80 °C), then disinfected with 1 % (v/v) MS Megades (Schippers, Bladel, The Netherlands) on the same day. The disinfection product consists of glutaraldehyde and quaternary ammonium compounds. After cleaning and disinfection, the pen remained vacant for 10 days. During this vacancy period, temperature and relative humidity (RH) were monitored hourly using thermo-hygrometers (Ilog EI-HS-D-32-L, ESCORT data logging systems). Three random pens per unit were sampled before disinfection and at 1, 4, 7 and 10 days after disinfection. Per sampling moment, 135 samples were taken, for a total of 675 samples.

### Sample processing

Sponge swab samples (3 M, SSL100, St. Paul, MN, USA), pre-moistened with 10 mL Ringers solution (Oxoid, BR0052G, Basingstroke, Hampshire, England), were taken at 5 locations per pen: floor, concrete wall, synthetic wall, drinking nipples and feeding trough. Sampling of 3 pens per unit resulted in triplicates per type of location or 15 swab samples per unit at each time point. To neutralise the residual action of the disinfectants on the microbiological growth, 10 mL Dey Engley neutralising broth (Sigma Aldrich, Fluka, D3435, St-Louis, MO, USA) was used to pre-moisten the sponge swab samples that were used on day 1 after disinfection. A surface of 625 cm^2^ (A4 paper format) was sampled whenever possible. Because the surface of the drinking nipples was smaller than 625 cm^2^, 2 drinking nipples per pen were sampled. Samples were transported to the lab under refrigeration and were processed immediately. For all measured pathogens, selected relevant parameters and enumeration or detection techniques were based on Luyckx et al. [12]. Swab samples were first diluted with 30 mL of Buffered Peptone Water (BPW, Oxoid, CM0509) and then homogenised by placing them in a Masticator (IUL instruments, S.A., Barcelona, Spain). Prior to plating, swab samples were further diluted in dilution series in saline peptone water (Bio Trading, K110B009AA, Mijdrecht, The Netherlands) to produce countable results on the selected agar media: Plate Count Agar (Oxoid, CM0325) for total aerobic flora and Slanetz and Bartley (Oxoid, CM0377) for *Enterococcus* spp. (lower enumeration limit 30 CFU/625 cm^2^). Plate Count Agar and Slanetz and Bartley plates were incubated at 30 and 37 °C during 72 and 48 h, respectively. A 10 mL BPW fraction was also transferred to a Stomacher® bag and mixed with 10 mL double concentrated Mueller Hinton Broth (Oxoid, CM0405) and 13 % (w/v) sodium chloride (Merck, 1.06404.500, Darmstadt, Germany). After overnight incubation of this solution at 37 °C, 100 μl was plated on chromID® MRSA SMART (MRSM, bioMérieux, Marcy l’Etoile, France) for the detection of MRSA. ChromID® MRSA SMART were incubated at 37 °C for 24–48 h. The remaining BPW fraction (original sample) was also incubated overnight at 37 °C for additional analyses: for detection of *E. coli* and faecal coliforms, 10 μl of the enrichment broth was plated onto Rapid *E. coli* medium (Biorad, 356–4024, Marnes-la-Coquettes, France) and incubated for 24 h at 44 °C.

### Statistical analysis

The distribution of the log-transformed enumerations of total aerobic flora and *Enterococcus* spp. was analysed via graphs (Q-Q plot and histogram). The log-transformed enumerations of total aerobic flora followed a normal distribution. A linear regression model was conducted to evaluate the effect of a vacancy period and location on the log-transformed total aerobic flora enumerations (dependent variable). To assess the effect of predictor variables (vacancy period and location) on the non-normally distributed outcome variables, variables describing the enumeration and detection of the different bacteria (*Enterococcus* spp., *E.* coli, faecal coliforms and MRSA) were transformed into binary variables (absent or below the detection limit = 0, present = 1). Subsequently a logistic regression analysis was carried out. Temperature and RH were added as covariates in both models. Variable “unit” was included as a random effect in both models to correct for measurements within one unit.

Post-hoc comparison was performed with a Tukey-Kramer test. *P*-values ≤ 0.05 were considered as significant. All statistical analyses were carried out using Statistical Analysis System software (SAS®, version 9.4, SAS Institute Inc., Cary, NC, USA).

## Abbreviations

0d: Day before disinfection
10d: 10 days after disinfection
1d: 1 day after disinfection
4d: 4 days after disinfection
7d: 7 days after disinfection
BPW: Buffered peptone water
C&D: Cleaning and disinfection
CFU: Colony forming units
*E. coli*: *Escherichia coli*
MRSA: Methicillin resistant *Staphylococcus aureus*
RH: Relative humidity
